# PRDI-BF1-RIZ domain of retinoblastoma protein-interacting zinc finger gene 1 induces apoptosis and exerts anticancer activity in esophageal squamous cell carcinoma cells

**DOI:** 10.3892/ol.2014.2671

**Published:** 2014-11-05

**Authors:** SHANG-WEN DONG, YAO-WEN ZHANG, YUAN CHEN, SHUO WANG, PEI SUN, YUAN-GUO WANG, PENG ZHANG

**Affiliations:** 1 Department of Cardiothoracic Surgery, Tianjin Medical University General Hospital, Tianjin 300052, P.R. China; 2 First Department of Radiotherapy, Henan Province Anyang Tumor Hospital, Anyang, Henan 455000, P.R. China; 3 Tianjin Institute of Endocrinology, Tianjin Medical University, Tianjin 300070, P.R. China

**Keywords:** retinoblastoma protein-interacting zinc finger gene 1, PRDI-BF1-RIZ domain, esophageal squamous cell carcinoma

## Abstract

The present study examined the role of the PRDI-BF1-RIZ (PR) domain of tumor suppressor retinoblastoma protein-interacting zinc finger gene 1 (*RIZ1*) as an anticancer domain and its ability to induce apoptosis in esophageal squamous cell carcinoma (ESCC) cells. The TE13 ESCC cell line was transfected with pcDNA3.1(+) eukaryotic expression vectors bearing the open reading frames of either the human *RIZ1* gene or the PR domain, and the mRNA and protein expression levels were then detected using quantitative reverse transcription polymerase chain reaction and western blotting, respectively. The rate of apoptosis was determined by flow cytometry and the cell invasion ability was determined by an invasion assay. RIZ1 and the PR domain induced apoptosis and reduced the cell invasion ability (P<0.01). These findings indicate that the *RIZ1* gene possesses anticancer activity in the PR domain, which may be important in inhibiting the development of ESCC.

## Introduction

The retinoblastoma protein-interacting zinc finger (*RIZ*) gene was identified by Bird (1) following the application of retinoblastoma (Rb) probes in combination with Rb protein to screen for separation-of-function mutants. Fluorescence *in situ* hybridization located the gene to human chromosome 1p36. Due to the presence of various transcription initiation sites, the *RIZ* gene encodes two proteins, RIZ1 and RIZ2. RIZ1 contains a positive regulatory (PR) domain, whereas RIZ2 does not; the sequences of the two proteins are otherwise identical (2). The PR domain, known as the PRDI-BF1-RIZ (positive regulatory domain I-binding factor 1-RIZ) homologous region, contains 100 amino acids that form the protein binding surface (protein-binding interface), mediating protein-protein interactions and exerting an important role in chromosome structure stability and in the regulation of chromatin gene expression (3). In tumors, the PR domain gene family expresses various protein products according to the presence or absence of the PR domain. A preponderance of either type of protein is indicative of gene inactivation, which is a predominant mechanism of tumorigenesis (4). Currently, a number of studies have demonstrated that *RIZ1* exerts tumor-inhibiting activity; for example, the RIZ1 protein may cause tumor cell arrest in the G_2_/M phase and induce apoptosis (5,6). In the present study, human *RIZ1* and PR domain eukaryotic expression vectors were constructed to investigate whether the PR domain of the tumor suppressor RIZ1 has the ability to induce apoptosis and reduce cell invasion ability in esophageal carcinoma cells.

## Materials and methods

### Cell culture and RNA isolation

The TE13 human esophageal squamous cell carcinoma (ESCC) cell line was purchased from American Type Culture Collection (Rockville, MD, USA). The cells were cultured in RPMI-1640 (Gibco-BRL, Carlsbad, CA, USA) containing 4.76 g HEPES, 2.0 g NaCO_3_, 10.4 g RPMI 1640 and 1,000 ml double-distilled (dd) H_2_O supplemented with 10% fetal bovine serum (FBS; Gibco-BRL), 1X L-glutamine (2 mm), 100 U/ml penicillin and 100 μg/ml streptomycin. The cells were incubated at 37°C in a 5% CO_2_ humidified incubator.

RNA was isolated from the cells using TRIzol (Invitrogen, Carlsbad, CA, USA) according to the manufacturer’s instructions, and 1 ml TRIzol was added to 5×10^6^-1×10^7^ cells. The RNA pellets were resuspended in diethylpyrocarbonate-treated H_2_O. The total RNA concentrations were quantified using an ultraviolet (UV) spectrophotometer (Beckman Coulter, Miami, FL, USA).

### Reverse transcription (RT) amplification of mRNA

RT reactions were performed to generate cDNA using 2 μg RNA, Moloney murine leukemia virus reverse transcriptase, ribonuclease inhibitor and a dNTP mixture (Takara Bio, Inc., Shiga, Japan), according to the manufacturer’s instructions. Semi-quantitative, RT-polymerase chain reaction (PCR) was conducted using the cDNA templates.

According to the *RIZ1* mRNA sequence published by the National Center for Biotechnology Information (NCBI), the 5,157 bp protein-coding region is located between base pairs 857 and 6,013. Due to the amplicon size, the open reading frame was divided into five sections, termed A603, A1200, B, C and D. The following primers were designed for the five *RIZ1* sections, hereafter referred to as amplicons, using Primer Premier 5.0 software (Premier Biosoft, Palo Alto, CA, USA): A603 forward, 5′-GTGGCTAGCATGAATCAGAACACTACTG-3′ and reverse, 5′-TTGGCTAGCAGAGGTGAAATCTGGCTC-3′; A1200 forward, 5′-TGGCTGCGATATGTGAATTG-3′ and reverse, 5′-CTCTACGCTGATGCCGTCTC-3′; B forward, 5′-GCTGATGGCAAAGCATCTG-3′ and reverse, 5′-AATTCCTTGCCTTCAGAGTCAC-3′; C forward, 5′-TCAAAGAAAGTCATTCAGTGC-3′ and reverse, 5′-CGGTGATGGTACTGAAATG-3′; and D forward, 5′-GCCTCAATCAGCATTACC-3′ and reverse, 5′-GTCTACTCTTTGAAGAATGGTC-3′. PCR was conducted in 50-μl reactions containing 5 μl 10X KOD buffer, 5 μl 2 mm dNTPs, 3 μl 25 mm MgSO_4_, 2 μl of each forward and reverse primer, 1 μl cDNA, 1 μl KOD-Plus-Ver. 2 polymerase (Toyobo Corporation, Osaka, Japan) and ddH_2_O. Each reaction required the following specific conditions in accordance with the melting temperature and size of each amplicon: Initial denaturation at 94°C for 2 min, 35 cycles of denaturation at 98°C for 10 sec, annealing (A603U, 60°C at 30 sec; A1200, 57°C at 30 sec; B, 55°C at 30 sec; and C and D, 50°C at 30 sec) and extension at 72°C for 1 min, and a final extension at 72°C for 10 min.

The PR domain primers were as follows: Forward, 5′-GTGGCTAGCATGAATCAGAACACTACTG-3′ and reverse, 5′-TTGGGATCCTCAAGAGGTGAAATCTG-3′. The 5′ terminal of the forward and reverse primers contained *Nhe*I and *Bam*HI endonuclease sites, respectively, and three protective base pairs. The downstream primer also contained a termination codon. Initial denaturation was performed at 94°C for 3 min, followed by 35 cycles of denaturation at 98°C for 10 sec, annealing at 60°C for 1 min and final extension at 72°C for 10 min. Subsequently, 0.3 μl Easy Taq (Tiangen, Beijing, China) was added to append a polyA tail at the end of the PCR products, turning the blunt end into a sticky end. This was followed by a final extension step at 72°C for 30 min.

The quality of the amplified products was analyzed on 12 g/l agarose gels using a UV spectrophotometer and the RT-PCR products were sequenced.

### Construction and transfection of the pcDNA3.1(+)/RIZ1 and pcDNA3.1(+)/PR domain

The amplicons were extracted from the agarose gels using the Tiangel Midi Purification kit (Tiangen, Beijing, China) according to the manufacturer’s instructions. The five *RIZ1* and PR domain amplicons were inserted into Trans1-T1 Phage Resistant vectors (Promega Corporation, Madison, WI, USA) that were subsequently transformed into Trans1-T1 Phage Resistant competent cells, and plated on agar containing ampicillin and X-gal. White colonies were selected for further analysis. Subsequent to expansion of the selected bacterial colonies, plasmid DNA was extracted by alkaline lysis (5). Restriction enzyme digests were employed to validate successful recombination, with confirmation provided by sequencing. The sequences of each plasmid were compared with the sequences listed by NCBI using the Basic Local Alignment Search Tool (http://blast.ncbi.nlm.nih.gov/Blast.cgi). The *RIZ1* and PR domain amplicons were digested from plasmids containing the correct insert and were ligated into the pcDNA3.1(+) eukaryotic expression vector (Invitrogen Life Technologies). Insertion was verified by restriction enzyme digestion followed by sequencing.

The TE13 cells were seeded in six-well culture plates at a density of 2×10^5^ cells/well in 2 ml media, and then incubated at 37°C to 90–95% confluence. After 24 h, the media was replaced with complete serum- or antibiotic-free RPMI-1640 in preparation for transfection. Ultra pure pcDNA3.1(+)/RIZ1 and pcDNA3.1(+)/PR domain plasmid DNA was extracted using a HighPure Mini Plasmid kit (Tiangen). A liposome-mediated method (6) was employed to transfect the TE13 cells with either the pcDNA3.1(+)/RIZ1 or the pcDNA3.1(+)/PR domain, with empty vector-transfected and untransfected cells serving as negative controls. Subsequent to 6 h of incubation with media containing the recombinant plasmids and the transfection reagents, the media was replaced with antibiotic-free RPMI-1640 containing 10% FBS. The transfected cells were incubated for a further 48 h, before being harvested for further analysis.

### Quantitative PCR

For the RNA isolation and reverse transcription reaction, 2μl of cDNA was mixed with 2X SYBR real-time PCR premixture (BioTeke, Beijing, China). The primers for the genes of interest (10μM) were as follows: RIZ1/PR domain forward, 5′-AATCAGAACACTACTGAGCCTGT-3′ and reverse, 5′-ACCAATCCGGGTCTTGTCAAC-3′; and glyceraldehyde-3-phosphate dehydrogenase (GAPDH) forward, 5′-GAAGGTGAAGGTCGGAGTC-3′ and reverse, 5′-GGGTGGAATCATATTGGAAC-3′. The reactions were conducted using an CFX96 Real-time PCR System (Bio-Rad, Hercules, CA, USA) according to the manufacturer’s instructions. Briefly, initial denaturation was performed at 95°C for 2 min, followed by 45 cycles of denaturation at 95°C for 15 sec, annealing for the RIZ1/PR domain at 63°C for 15 sec or for GAPDH at 60°C for 15 sec, and extension at 72°C for 40 sec, followed by the production of thermal melting curves. Each sample for each gene was conducted in triplicate.

### Western blotting

The TE13 cells transfected with pcDNA3.1(+)/RIZ1 were homogenized in radioimmunoprecipitation buffer (containing 50 mm Tris-HCl, pH 7.4; 150 mm NaCl; 1% Nonidet P-40; 0.5% sodium deoxycholate; 0.1% SDS; 1 mm EDTA; 1 mm phenylmethylsulfonyl fluoride and 1 mg/ml aprotinin) and the protein concentrations were determined using a bicinchoninic acid protein assay kit (Pierce, Rockford, IL, USA). Cell lysates (30 μg) were separated by 8% SDS-PAGE, transferred to nitrocellulose membranes (Amersham Biosciences, Chalfont St. Giles, UK) and immunoblotted with the indicated antibodies overnight in the Orbital Shaker (Thermo Fisher Scientific, Waltham, MA, USA) at 4°C. The monoclonal mouse anti-human RIZ1/PR domain antibodies, monoclonal mouse anti-human β-actin primary antibody and secondary polyclonal goat anti-mouse polyclonal antibody were all obtained from Abcam (Cambridge, UK).

Bands were visualized using a PowerLook scanner (UMAX Technologies, Hsinchu, Taiwan) and quantified using ImageQuant software (GE Healthcare, Little Chalfont, UK). The relative expression levels of RIZ1 and the PR domain were calculated as the gray values of RIZ1, the PR domain and β-actin. Untransfected and empty vector-transfected TE13 cells served as negative controls.

### Flow cytometric analysis

To investigate the effect of overexpression of RIZ1 or the PR domain on apoptosis, TE13 cells were seeded in six-well plates at a density of 2×10^5^ cells/well and allowed to attach for 12 h. The cells were then transfected with either pcDNA3.1(+)/RIZ1 or pcDNA3.1(+)/PR domain and harvested after 24 h. A total of ~1×10^5^ cells were washed with cold phosphate-buffered saline (PBS; Solomen, Tianjin, China) for each test. Cells were suspended in 1 ml 1X binding buffer using the Annexin V-fluorescein isothiocyanate (FITC) Apoptosis Detection Analysis Kit (Tianjin Sungene Biotech Co., Ltd., Tianjin, China). Next, the cells were centrifuged (5415D; Ruicong Co., Ltd., Shanghai, China) at 300 × g for 10 min at room temperature and the supernatant was removed. The cells were then resuspended in 1 ml 1X binding buffer and the cell concentration was adjusted to 1×10^6^ cells/ml. A total of 100 μl cell suspension was used for analysis. Next, 5 μl Annexin V-FITC staining solution was added and the tube was kept in the dark at room temperature for 10 min. A total of 5 μl propidium iodide staining solution (Tianjin Sungene Biotech Co., Ltd.) was added and the tube was kept in the dark at room temperature for 5 min. Finally, 500 μl PBS was added to the cells and vortexed gently for 1 h. The cells were then analyzed using a BD FACSAria II cell sorter (BD Biosciences, Franklin Lakes, NJ, USA). Untransfected and empty vector-transfected TE13 cells served as negative controls.

### Cell Invasion Assay

To study the invasion ability using a cell invasion assay kit (ECM550; Millipore, Billerica, MA, USA). The invasion chamber was allowed to adjust to room temperature in a tissue culture hood. Media containing 10% FBS (500 μl) was added to the lower chamber and 300 μl of cell suspension, containing 1.0×10^6^ cells/ml in serum-free media, was added to each insert. The invasion assay was then incubated for 24 h in a tissue culture incubator. Using a cotton-tipped swab, the non-invading cells were then gently removed, and the ECMatrix gel was also removed from the interior of the inserts. The invasive cells on the lower surface of the membrane were stained by dipping the inserts in the crystal violet staining solution for 20 minutes and then dipping the inserts in a beaker of water three times to rinse. The cells were counted by capturing images of the membrane through the microscope (DFC480, Leica, Wetzlar, Germany).

### Statistical analysis

Statistical analysis was conducted using SPSS 18.0 software (SPSS, Inc., Chicago, IL, USA). The data are presented as the mean ± SD. The quantitative PCR results are shown as 2^−averageΔΔCT^ × 100%. Student’s t-test and one-way analysis of variance (ANOVA) were employed to examine parametric data. The χ^2^ test was used for statistical analysis of the group comparisons and to compare enumerated data. P<0.05 was considered to indicate a statistically significant difference.

## Results

### Expression levels of RIZ1 and PR domain following pcDNA3.1(+)/RIZ1 and pcDNA3.1(+)/PR domain transfection

**Figure 1:**
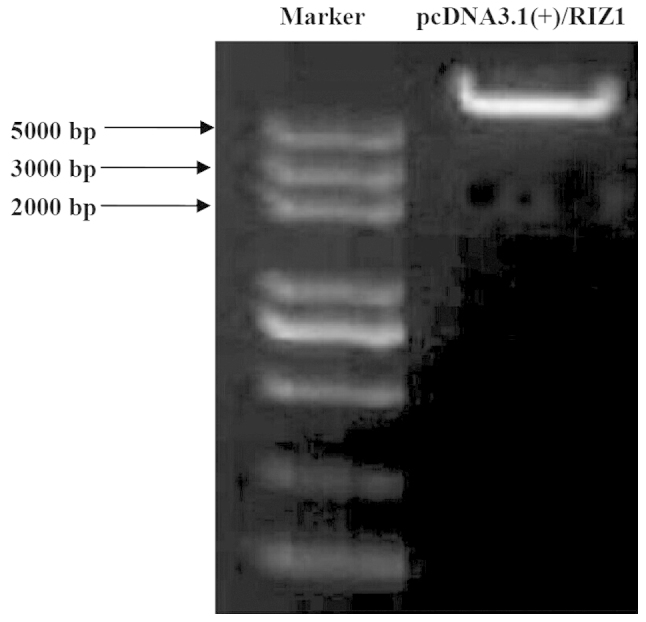
A603, A1200, B, C and D segments were ligated into pcDNA3.1(+) and verified by restriction enzyme digestion. The appearance of the 10,585 bp pcDNA3.1(+)/RIZ1 band was consistent with the expected results. RIZ1, retinoblastoma protein-interacting zinc finger protein 1.

**Figure 2:**
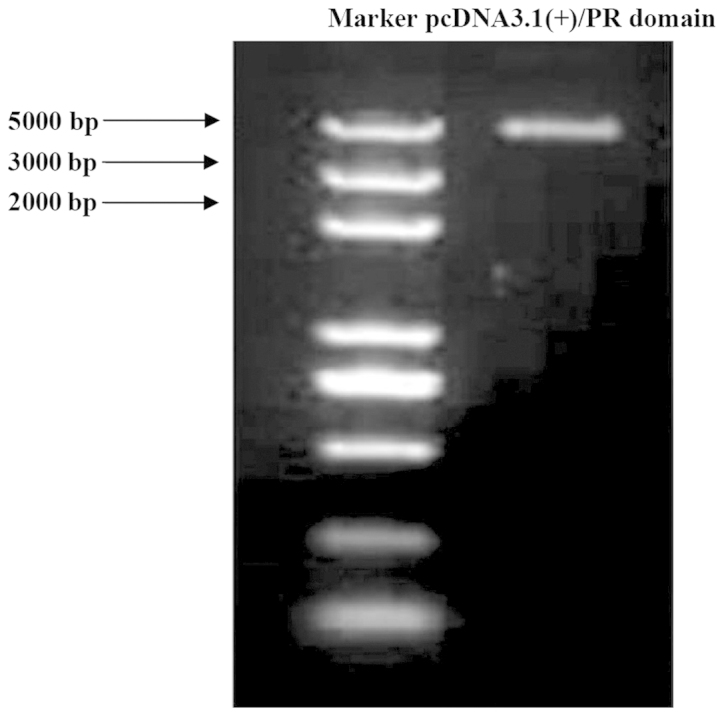
Agarose gel electrophoresis of pcDNA3.1(+)/PRDI-BF1-RIZ (PR) domain polymerase chain reaction product. The appearance of the 6031 bp band was consistent with the expected results.

To overexpress *RIZ1* and the PR domain, recombinant plasmids were generated to enable ectopic overexpression of *RIZ1* (Fig. 1) and the PR domain (Fig. 2) in TE13 cells.

### Quantitative RT-PCR and western blotting

**Figure 3:**
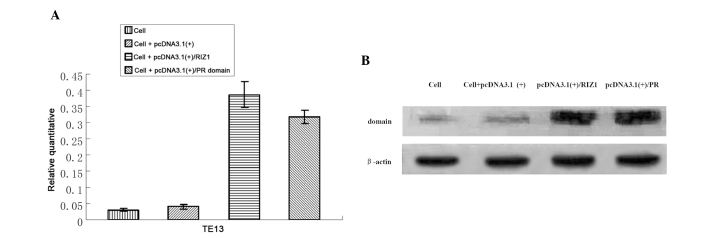
(A) Quantitative reverse transcription polymerase chain reaction analysis of *RIZ1* and PR domain mRNA expression levels in TE13 esophageal squamous cell carcinoma cells transfected with either pcDNA3.1(+)/RIZ1 or pcDNA3.1(+)/PRDI-BF1-RIZ (PR) domain. The *RIZ1* and PR domain mRNA expression levels were normalized to those of β-actin. The difference in expression levels between the untransfected and empty vector-transfected cells (negative controls) was not statistically significant (P>0.05). Significantly higher RIZ1 and PR domain mRNA expression levels were detected in cells transfected with pcDNA3.1(+)/RIZ1 or pcDNA3.1(+)/PR domain (P<0.01). (B) Western blot analysis of RIZ1 and PR domain protein expression levels in TE13 cells transfected with either pcDNA3.1(+)/RIZ1 or pcDNA3.1(+)/PR domain. β-actin served as a loading control and untransfected and empty vector-transfected TE13 cells served as the negative control. Significantly higher RIZ1 and PR domain protein expression levels (P<0.01) were detected in cells transfected with pcDNA3.1(+)/RIZ1 or pcDNA3.1(+)/PR domain compared with negative controls.

The RIZ1 and PR domain mRNA and protein expression levels in TE13 cells transfected with pcDNA3.1(+)/RIZ1 or pcDNA3.1(+)/PR were significantly higher compared with untransfected and empty vector-transfected cells (negative control groups) (P<0.05; Fig. 3). No statistically significant differences were identified between the negative control groups (P>0.05).

### Flow cytometric analysis of the apoptotic rate in pcDNA3.1(+)/RIZ1 or pcDNA3.1(+)/PR domain-transfected TE13 cells

**Figure 4:**
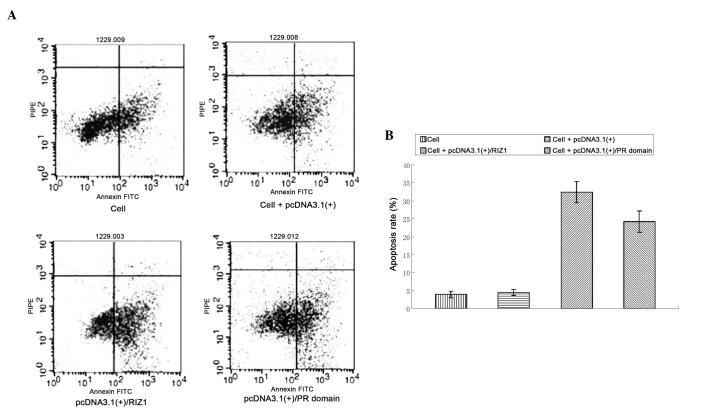
Flow cytometric analysis of apoptosis in TE13 esophageal squamous cell carcinoma cells transfected with either pcDNA3.1(+)/RIZ1 or pcDNA3.1(+)/positive regulatory (PR) domain. (A) Representative flow cytometric plots. (B) The proportion of apoptotic cells was significantly higher in cells transfected with pcDNA3.1(+)/RIZ1 or pcDNA3.1(+)/PR domain (P<0.01) than in negative controls.

The apoptotic rates were significantly higher in the cells transfected with pcDNA3.1(+)/RIZ1 or the pcDNA3.1(+)/PR domain compared with the untransfected and empty vector-transfected TE13 cells (P<0.01; Fig. 4).

### Cell Invasion Assay

**Figure 5:**
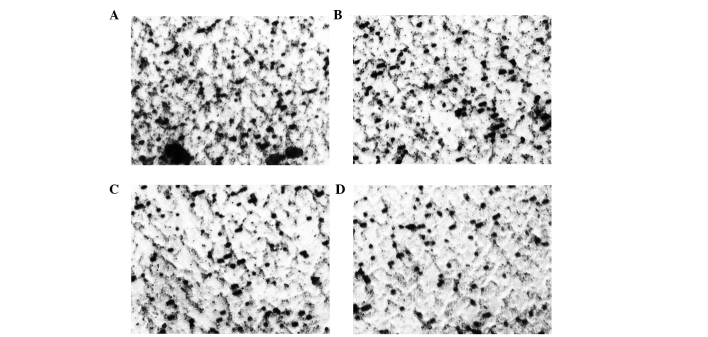
The Matrigel invasion ability of (A) untransfected cells (124.71±12.81 cells/HP), (B) empty vector transfected cells (125.62±8.57 cells/HP), (C) pcDNA3.1(+)/RIZ1 cells (82.65±12.79 cells/HP), (D) pcDNA3.1(+)/PR domain cells(83.97 ± 12.83 cells/HP). The Matrigel invasion ability of the TE13 cells transfected with pcDNA3.1(+)/RIZ1 and pcDNA3.1(+)/PR-domain was significantly reduced compared with the negative controls (P<0.05). Cells stained with crystal violet. Magnification, ×200.

The Matrigel invasion ability of the TE13 cells transfected with pcDNA3.1(+)/RIZ1 or pcDNA3.1(+)/PR domain was significantly reduced compared with the invasion ability of the negative controls (P<0.05; Fig. 5).

## Discussion

The silencing of tumor suppressor genes (TSGs) by genetic and epigenetic pathways is recognized as important in human carcinogenesis (7,8). In addition to gross chromosomal instability and the instability of small repetitive DNA sequences, epigenetic silencing is also considered to contribute significantly to human carcinogenesis. TSG silencing by the methylation of CpG-rich promoter regions has been reported in numerous types of human cancer. The distal region of the short arm of human chromosome 1 (1p36) is commonly deleted in a variety of human tumor types. This region is known to harbor several TSGs. One candidate TSG in this region is *RIZ1* (9).

The *RIZ* gene encodes two protein products of different lengths, RIZ1 and RIZ2. As a TSG, *RIZ1* contains the PR or Suvar3–9, Enhancer-of-zeste, Trithorax domain, but RIZ2 lacks this domain. A number of studies have investigated the underlying mechanism of *RIZ1* gene inactivation, which has been shown to include genetic and epigenetic changes (10–12). Chromosomal and microsatellite instability deactivates the RIZ1 gene, resulting in frameshift mutation, point mutations and heterozygous deficiency (13). Silenced or reduced expression levels of the RIZ1 gene have been observed in numerous types of human tumor and tumor cell lines; however, to the best of our knowledge, no study of the RIZ1 gene in esophageal cancer has been reported.

Previously, quantitative RT-PCR was performed and the RIZ1 mRNA expression levels in esophageal cancer were found to be significantly lower compared with those in normal esophageal tissue, and this was associated with CpG island methylation (14,15). This indicated that inactivation of RIZ1 may be important in the progression of esophageal cancer.

The ability of the PR domain alone to exert any anticancer activity was examined, as the PR domain is the only structural difference between the two protein products of the RIZ gene. Compared with RIZ2, RIZ1 possesses an additional 100 amino acid residues at the amino terminus, and the PR domain represents the main functional motif within the amino terminus of RIZ1. In the present study, TE13 cells were transfected with pcDNA3.1(+)/PR domain to investigate whether the domain could be expressed independently and whether it influenced apoptosis or invasion. Quantitative PCR and western blotting determined that cells transfected with the recombinant plasmid successfully expressed RIZ1 and the PR domain. Flow cytometry revealed that transfection with either the PR domain or RIZ1 inhibited cell proliferation. The cell invasion assay revealed that the invasion ability was significantly reduced in the TE13 cells transfected with pcDNA3.1(+)/RIZ1 or pcDNA3.1(+)/PR-domain (P<0.05). These findings indicate that the PR domain of RIZ1 exerts anticancer activity in ESCC. In conclusion, further study regarding the mechanism of action of the RIZ1 tumor suppressor gene and the PR domain may reveal the underlying mechanism of anticancer function, and thus may lead to the development of novel biomarkers for early diagnosis and prognostic evaluation in ESCC.
